# Multimodal Characterization of Neural Networks Using Highly Transparent Electrode Arrays

**DOI:** 10.1523/ENEURO.0187-18.2018

**Published:** 2018-01-10

**Authors:** Mary J. Donahue, Attila Kaszas, Gergely F. Turi, Balázs Rózsa, Andrea Slézia, Ivo Vanzetta, Gergely Katona, Christophe Bernard, George G. Malliaras, Adam Williamson

**Affiliations:** 1 Department of Bioelectronics, Ecole Nationale Supérieure des Mines, Centre of Microelectronics in Provence, Gardanne 13541, France; 2 Aix Marseille Univ, CNRS, INT, Inst Neurosci Timone, Marseille, France; 3 Department of Psychiatry, Division of Systems Neuroscience, Columbia University and Research Foundation for Mental Hygiene, New York State Psychiatric Institute, New York, NY 10032; 4 Laboratory of 3D Functional Network and Dendritic Imaging, Institute of Experimental Medicine, Hungarian Academy of Sciences, Budapest H-1083, Hungary; 5 Aix Marseille Univ, INSERM, INS, Inst Neurosci Syst, Marseille, France; 6 Neuroengineering Research Group, Interdisciplinary Excellence Center, Department of Medical Microbiology and Immunobiology, University of Szeged, Szeged 6720, Hungary; 7 Two-Photon Measurement Technology Research Group, Pázmány Péter Catholic University, Budapest H-1083, Hungary; 8 Electrical Engineering Division, Department of Engineering, University of Cambridge, Cambridge CB3 0FA, United Kingdom

**Keywords:** electrophysiology, neuroengineering, organic electronics, PEDOT:PSS, transparent electronics, two-photon imaging

## Abstract

Transparent and flexible materials are attractive for a wide range of emerging bioelectronic applications. These include neural interfacing devices for both recording and stimulation, where low electrochemical electrode impedance is valuable. Here the conducting polymer poly(3,4-ethylenedioxythiophene):poly(styrenesulfonate) (PEDOT:PSS) is used to fabricate electrodes that are small enough to allow unencumbered optical access for imaging a large cell population with two-photon (2P) microscopy, yet provide low impedance for simultaneous high quality recordings of neural activity *in vivo*. To demonstrate this, pathophysiological activity was induced in the mouse cortex using 4-aminopyridine (4AP), and the resulting electrical activity was detected with the PEDOT:PSS-based probe while imaging calcium activity directly below the probe area. The induced calcium activity of the neuronal network as measured by the fluorescence change in the cells correlated well with the electrophysiological recordings from the cortical grid of PEDOT:PSS microelectrodes. Our approach provides a valuable vehicle for complementing classical high temporal resolution electrophysiological analysis with optical imaging.

## Visual Abstract

**Figure:**
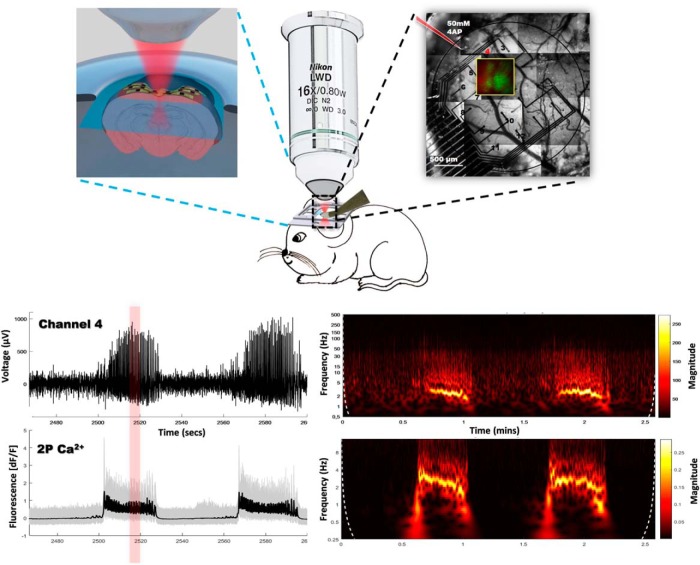

## Significance Statement

Electrophysiological recordings, with varying degrees of invasiveness, are the traditional method for measuring neural activity, possessing the capability to measure both individual neurons and populations of neurons. Imaging methods, such as computed tomography scans and functional magnetic resonance imaging, have been developed to accomplish less invasive characterization of neuronal activity; traditionally offering good spatial or relatively high temporal resolution, yet resolution of individual neurons cannot be achieved. Two-photon (2P) imaging enables network-wide analysis with cellular resolution on a faster timescale with high spatial fidelity. This study presents a method for the combination of 2P imaging and electrophysiological recordings with highly transparent arrays of organic electrodes, presenting a powerful tool to simultaneously acquire the electrical and optical activity of neural circuits.

## Introduction

Network-level brain functions result from the activity of large populations of neurons. Optical and electrophysiological techniques have been developed to interrogate the functions of the interconnected neurons within networks and to track the activity of as many individual neurons as possible. High-density neural probes for electrophysiological applications record hundreds of neurons simultaneously. This technique allows for the direct assessment of individual neuronal activity (firing of action potentials) as well as population activity of many neurons (field potentials; Buzsáki and Draguhn, 2004). The main disadvantage of electrophysiological recordings is poor spatial resolution (Taketani and Baudry, 2010). For example, implantable probe geometry does not allow the recording of all neurons belonging to one functional unit, such as a cortical column. Calcium (Ca^2+^) imaging records large populations of neurons with high spatial resolution, offering an option to overcome this disadvantage (Stosiek et al., 2003). The Ca^2+^ signal, however, is an indirect measure of cellular activity, thus it would be ideal to combine physiologic and optical techniques. To achieve this combination, the electrophysiological signal should be measured where the optical signal is obtained. This approach requires a highly transparent electrophysiological device to allow efficient collection of the optical signal. Although recent developments for this scenario have been demonstrated *in vitro* (Chang et al., 2011; Benfenati et al., 2013; Scott et al., 2013)_,_ the simultaneous acquisition of neural activity *in vivo* has remained a technical challenge.

Studies aiming to combine *in vivo* single cell electrophysiological recordings with 2P imaging use intracellular or extracellular glass electrodes (Helmchen et al., 2001; Svoboda and Yasuda, 2006; Kitamura et al., 2008) employing a Ag/AgCl electrode to close the recording circuit. Alternatively, a metal electrode may be chronically implanted in the contralateral hemisphere, while the image collection is conducted at physical distance (Malvache et al., 2016), a method which assumes high correlation between the two recording sites. These methods have limitations including use of delicate recording pipettes, limited number of recording sites and the necessity to locate the metal parts of the electrode outside the field of view as it is prone to generate noise due to the photoelectric effect (Kozai and Vazquez, 2015). Flexible materials and micro-fabrication techniques have been explored over recent years to solve the former two limitations. The photoelectric effect, however, complicates the simultaneous use of optical and electrophysiological signal recording for a great deal of materials and engineering approaches. An *in vitro* study by Kuzum et al. (2014) well demonstrated the possibility to image hippocampal slices using confocal and 2P techniques through a flexible graphene electrode array, benefitting from the high-transparency of the electrode material and interconnecting lines. The used Kapton substrate, however, somewhat hinders the overall optical transparency and the 50 × 50 µm electrode surface has high electrochemical impedance (∼500 kΩ at 1 kHz), the main requirement for capturing high-quality electrophysiological signals (Franks et al., 2005; Williams et al., 2007). In a separate study on flexible graphene electrodes, a high level of transparency was exhibited by Park et al. (2014) using a parylene C (PaC) substrate. Fluorescence imaging through the array was possible *in vivo*, as well as electrophysiological signal recording and optogenetic stimulation of the underlying tissue, however, relatively large electrodes are used (∼150 µm in diameter) with 1-kHz impedance values reduced only to ∼250 kΩ. Recent work has pushed toward improved technologies offering *in vivo* electrophysiology and optical recording capabilities. These reports include arrays of 100 × 100 µm graphene electrodes (963-kΩ average impedance) with high transparency (Thunemann et al., 2018) as well as a study by Qiang et al. (2018) using a gold nano-mesh patterning technique, creating electrodes with diameters down to 20 µm with an average 1-kHz impedance value of 130.3 kΩ, employing a similar material system to ours. Main differences include: insulation material (SU8 vs PaC), overall higher electrode impedance (∼5×) and device thickness (∼4×), but improved transparency at the mesh’s conduction lines and recording sites.

In this report, we demonstrate an extremely flexible (∼4-µm-thick) cortical microelectrode array (25 × 25 µm electrode size), with moderate channel density deposited on an optically transparent PaC substrate. The high overall transparency in combination with low impedance values (∼25 kΩ at 1 kHz) enables electrophysiological and optical recordings from the same location. To achieve low impedance, we employ the well-known ionic/electronic conducting polymer poly(3,4-ethylenedioxythiophene):poly(styrenesulfonate) (PEDOT:PSS). PEDOT:PSS is applied at the electrode sites, efficiently reducing the impedance and allowing for minimization of the contact lines and electrode area. With this approach, unobstructed imaging may be performed through >95% of the substrate area intended for optical and electrical measurement. The micron-scale organic electrode grid maximizes the overall optical transparency and allows for simultaneous 2P imaging of the underlying tissue.

## Materials and Methods

### Device fabrication

The organic electrode grid fabrication process is based on previously reported methods (Khodagholy et al., 2013, Sessolo et al., 2013). An initial PaC film was deposited on glass slides using a SCS Labcoater 2 with a resulting thickness of 2 µm. Once the subsequent fabrication steps are completed, this PaC layer is delaminated from the glass, thus acting as the final substrate and providing the flexibility for the Electrocorticography (ECoG) array. Photolithography and lift-off processes were employed to pattern metal interconnects on top of the PaC substrate. This was performed using a positive photoresist (Shipley MICROPOSIT™ S1813), a SUSS MJB4 UV broadband contact aligner and MF26 developer. A 2-nm chromium adhesion promoting layer and 100 nm of gold were thermally evaporated onto the substrates and the samples were immersed in an acetone bath to define the interconnects through lift-off. To electrically insulate the metal lines, a second PaC film was deposited on the devices to thicknesses of 2 µm, using the same deposition process as before, however with 3-(trimethoxysilyl)propylmethacrylate present in the chamber (by addition of a droplet to the chamber wall) to act as an adhesion promoter. Subsequently, a dilute solution of Micro-90 industrial cleaner was spin coated onto this insulation layer, followed by the deposition of a sacrificial PaC layer (2 µm). These steps allow the sacrificial layer to later be peeled off from the substrate, defining PEDOT:PSS at the electrode recording sites. The PaC layers were etched by reactive ion etching (RIE) in an Oxford 80 Plasmalab plus with an O_2_/CHF_3_ plasma to open the electrode sites by creating an opening down to the gold interconnect. AZ9260 photoresist was used as an etch mask, patterned using the UV contact aligner and AZ developer. A dispersion of PEDOT:PSS (CleviosTM PH 1000 from Heraeus Holding GmbH), 5 volume % ethylene glycol, 0.1 volume % dodecyl benzene sulfonic acid, and 1 wt % of (3-glycidyloxypropyl)-trimethoxysilane was spin coated onto the substrates to attain a thickness of 200 nm. The sacrificial PaC layer was peeled off removing superfluous PEDOT:PSS and defining the electrode sites. The devices were baked for 1 h at 140°C to crosslink the film. Finally, the devices were immersed in deionized water (DIW) to remove low molecular weight compounds. This DIW soak also facilitates the delamination of the final flexible electrode array from the glass slide.

### Electrophysiological signal acquisition and processing

Neural data were recorded using a RHD2132 Intan technology amplifier board. Zero insertion force clips (ZIF-Clip) were used to connect directly to the ECoG devices, along with an adaptor chip to connect to the Intan technology head stage. A sampling rate of 20 kHz was used and data were stored in 16-bit format. Data analysis was conducted using MATLAB (MathWorks). A 400 Hz low pass filter was applied to the acquired signal.

### Surgical procedure

Transgenic Thy1-GCaMP6f mice (Dana et al., 2014) with neurons expressing a fluorescent calcium indicator (14–16 weeks old, three males) were housed according to institutional regulations in the animal facilities of corresponding universities. On the day of surgery, animals were sedated with 2% isoflurane, then fixed in a stereotactic frame and kept under 4% sevoflurane anesthesia. After a subcutaneous lidocaine injection, the skull was exposed and a cranial window (4 mm in diameter) was drilled above the somatosensory cortex. A head plate was mounted onto the skull by dental acrylic, and the animal was placed into the microscope setup using the head plate. The temperature of the mouse was monitored and controlled using a rectal probe and heated mouse pad set to 38°C. During the recording procedure the anesthesia was maintained with a 1.2% isoflurane and oxygen mixture. The surface probe was positioned onto the craniotomy and covered with artificial cerebrospinal fluid, containing: 124 mM NaCl, 26 mM NaHCO_3_, 3.5 mM KCl, 1 mM MgCl_2_, 1 mM CaCl_2_, and 10 mM D-glucose. A coverslip was gently placed on top of the surface probe, and a tile map was acquired by an infrared CCD camera through a 4× objective. 4-Aminopyridine (4AP) was used to evoke seizure-like activity in the cortex. The drug was pressure injected via a glass pipette containing 50 mM 4AP with 20 µM Alexa Fluor 594 as a red dye for marking the injection site. The drug injection was positioned underneath both the cover glass and the probe ∼500 µm away from the imaging field of view, at a depth of 500 µm, using a micromanipulator (PatchStar, Scientifica). All procedures were approved by the Aix-Marseille Université ethical committee and were in accordance with guidelines of the corresponding institutes.

### 2P imaging

At the beginning of the experiments, a reference z-stack of the volume was acquired on a dual-scanhead 2P microscope (FemtoS-Dual, Femtonics Ltd) equipped with a femtosecond pulsed laser tuned to 920 nm (Mai Tai HP, SpectraPhysics) using a Nikon LWD 16x/0.8 NA objective. A single acquisition plane was selected and full-frame imaging was started in resonant scanning mode at 30.9375 Hz. The control of imaging and the trigger of electrophysiological recordings was done using the microscope’s acquisition software (MESc). We used the MESc and the MES software packages (Femtonics Ltd) to analyze imaging data (Katona et al., 2012).

## Results

**Figure 1.**
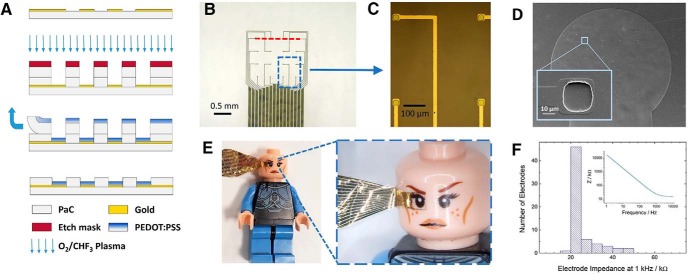
Organic cortical electrode grid. ***A***, Cross-sectional schematic of the microfabrication process corresponding to the area in ***B*** indicated by the red dotted line. Note: illustrative view is not to scale ***B***, Top view of the electrode array. ***C,*** Microscopic image of individual organic PEDOT:PSS electrode sites in the area of the array indicated by the blue square in ***B***. ***D***, Scanning electron microscope image of the full ECoG array with a zoomed-in view of one recording site in the inset. ***E***, Eye of a Lego toy clearly visible through the device (transparent area intended for imaging centered on the eye; note the bundle of contact lines visible extending to the left of the toy). ***F***, Histogram of 64 electrode impedances from four devices at 1 kHz with an example electrochemical impedance spectrum in the inset.

The developed organic electrode device, produced through the microfabrication steps described in the Materials and Methods section, is presented in Figure 1. Figure 1*A* shows the schematic outline of the fabrication process. The dashed line in Figure 1*B* corresponds to the cross-sectional area shown in Figure 1*A*. Optical images of the resulting grid of organic electrodes are given in Figure 1*B*,*C*. Overall, the micro-electrode grid consists of gold lines and 16 PEDOT:PSS recording sites patterned on a flexible PaC substrate. The interconnects are insulated using a secondary layer of PaC, resulting in a thin (∼4 µm) overall final device, extremely conformable to the surface of the brain. Openings etched in a third sacrificial layer of PaC (and through the insulation layer) are used to selectively pattern PEDOT:PSS to the desired electrode sites through a previously developed peel-off method (Khodagholy et al., 2013; Sessolo et al., 2013). Figure 1*B* shows the resulting 16-electrode array patterned on a 2.5-mm diameter PaC substrate area. Each electrode recording site is 25 × 25 µm, with an interelectrode spacing of 400 µm. A close-up view of a representative area indicated in blue is shown in Figure 1*C*, with the conducting polymer coating visible on four electrode sites. A scanning electrode microscope (SEM) image of the entire eletrode array is shown in Figure 1*D*, with a zoomed-in view of one electrode in the inset. Utilization of PEDOT:PSS at the recording sites is integral for this probe, making it possible to maintain low electrochemical electrode impedance. The 1-kHz impedance values of 64 electrodes from four devices are shown in Figure 1*F*, all below 50 kΩ with an average impedance of 25.8 kΩ; a value very well suited for electrophysiological recordings. The possibility to attain this impedance range with electrodes and interconnects on the tens of micrometers range allows for maximizing the area of transparent substrate which can be used for imaging. The transparency facilitates straightforward alignment and orientation of the array with biological structures below the grid. Figure 1*E* demonstrates these features, with the electrode grid placed on one eye of a Lego toy figure.

**Figure 2.**
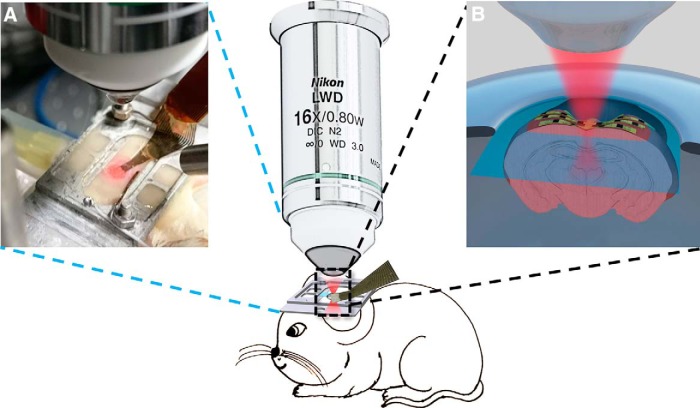
Layout of the recording setup with 2P-compatible electrode array. Head-fixed, anesthetized animal under the 2P microscope with the grid of organic electrodes in place, monitoring electrophysiological activity of the cortex. ***A***, Photo of anesthetized animal’s headplate with the probe positioned on the cortex under the objective. ***B***, The infrared laser beam (pseudo-color red) can pass through the grid of organic electrodes, as well as the subsequent visible emitted fluorescence, measured at a depth of up to 1mm into the tissue, monitoring activity across the entire scanned plane (blue). Cortical electrophysiological recordings are then compared to the calcium signal measured by the 2P system for dual characterization of the neural network activity.

**Figure 3.**
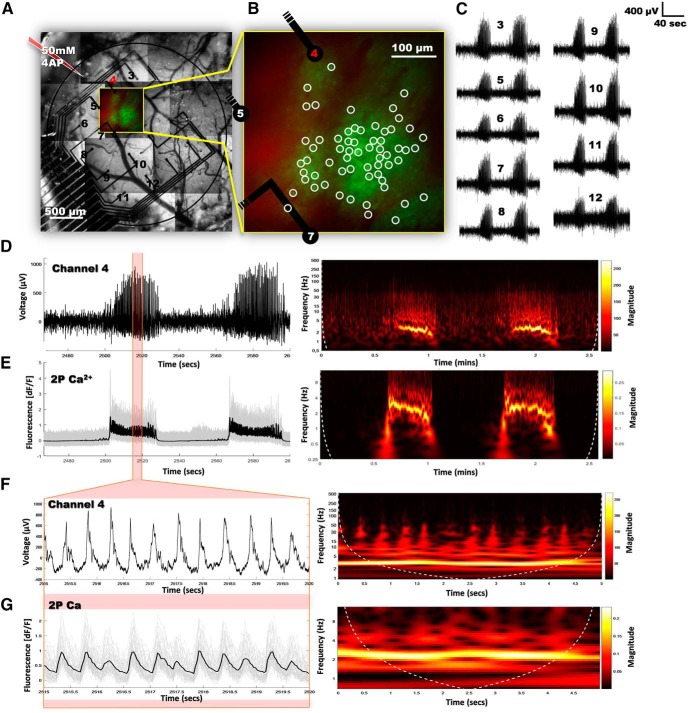
Simultaneous *in vivo* 2P imaging and cortical electrophysiological recording. ***A***, Infrared camera picture montage of the flexible 16-channel cortical electrode array positioned in a craniotomy above the primary visual cortex of a Thy1-GCaMP6f mouse. ***B***, Average image of 60 frames from a 2P time sequence measurement of GCaMP6f (green) and Alexa Fluor 594 (red) labeling. The selected cells (*n* = 63, white circles) were located 200 µm below the cortical surface. Electrode locations in this area are indicated. ***C***, LFP recordings from channels depicted in ***A***. ***D***, ***E***, Simultaneous LFP recording (D, left) and calcium imaging (E, left) from single cells (gray) and the average (black) of interictal and ictal periods evoked by 50 mM 4-AP injection. Right panels, Corresponding continuous 1-D wavelet transform analysis. ***F***, ***G***, Zoomed-in view of ictal events on LFP (F, left) and calcium traces (G, left) for single cells (G, gray) or the population average (G, black). Right panels, Corresponding continuous 1-D wavelet transform analysis.

As seen in Figure 2, the implemented grid of electrodes with 16 recording sites is placed on the surface of the cortex, directly in the path of the 2P scan. This provides multiple sites for electrophysiological measurements during 2P calcium imaging, representing a significant advantage over a single electrophysiological recording site of a glass pipette. Figure 2*A*,*B* shows the layout of the experimental setup. The anesthetized mouse was head-fixed in the 2P setup and the grid of organic electrodes was placed over the visual cortex. The flexibility of the organic electrodes and the substrate allows for continuous contact with the brain surface, ensuring a good electrochemical interface between the tissue and the probe. The surface probe was covered with a glass cranial window carefully aligned to leave ample space for an entry point of a glass injection pipette used to deliver 4-AP (50 mM), a well-known method to induce epileptiform activity (Voskuyl and Albus, 1985; Szente and Baranyi, 1987; Avoli et al., 2002). During 2P imaging sessions, the emitted green fluorescence of the neuronal calcium indicator under the flexible grid was recorded simultaneously with the electrophysiological activity (Fig. 3).

The orientation of the flexible grid of organic electrodes on the surface of the cortex is shown in Figure 3*A*. The inset of Figure 3*A* and zoomed view in Figure 3*B* show the average of 60 frames from a 2P time sequence measurement, imaged through the electrode grid 200 µm below the cortical surface. Neurons here expressed GCaMP6f (green) as a calcium indicator, while Alexa Fluor 594 (red) labeling as a fluorescent marker was used to follow the spread of the 4-AP solution after injection. While Figure 3*A* numbers all available electrode sites used for recording, the numbering indicated in Figure 3*B* shows those electrodes that were located in the 2P field-of-view. Electrophysiological local field potential (LFP) recordings for nearby electrodes are shown in Figure 3*C*, with a zoomed view of electrode 4 in Figure 3*D*, as this electrode is the most relevant regarding spatial proximity. The data represent an example of seizure activity within the tissue induced by an injection of 50 mM 4-AP (injection pipette shown in Fig. 3*A*). The LFP recording of electrode 4 is compared with 2P calcium imaging data in Figure 3*D–G*. Fluorescence changes, arising from fluctuations in intracellular calcium concentrations (Fig. 3*E*, left), clearly correspond to the simultaneously recorded electrophysiological fluctuations (Fig. 3*D*, left). Calcium imaging data are shown for single cells (gray, *n* = 63) as well as the average of the population response (black). Figure 3*D*,*E*, right panels, shows the corresponding continuous 1-D wavelet transform analysis, for the frequency analysis of the average responses. Figure 3*F*,*G* presents a zoomed-in view of the area indicated in red in Figure 3*D*,*E*. On this timescale ictal events on LFP (Fig. 3*F*) and calcium traces (Fig. 3*G*) for both single cells (gray traces) and the population average (black trace) are also very well correlated. These measurements demonstrate the simultaneous monitoring of neural activity both optically and electrically, and open further possibilities for a wide variety of studies to be explored in the future.

## Discussion

The aim of this work was to demonstrate the feasibility to record optical and electrophysiological signals for the same site simultaneously *in vivo*. Previously, this has been problematic due to the highly reflective inorganic materials used to create electrodes and the photoelectric effect caused by the laser light beam at conduction lines and recording sites. We minimized these issues by making use of an organic array of electrodes on an optically transparent substrate. To accomplish this, a low impedance PEDOT:PSS-based microelectrode array was developed (Fig. 1) to ensure that a high level (>95%) of the area of interest remains unobstructed for laser scanning, allowing for simultaneous fluorescence signal collection through 2P microscopy (Fig. 2). The mixed conduction PEDOT:PSS material system has received a great deal of attention due to its excellent electronic and mechanical properties, and the resulting practicality and commercial availability (Elschner et al., 2011)_._ The material flexibility is an additional benefit, providing excellent contact with the cortical surface and reduced invasiveness compared to traditionally stiffer implantable devices (Khodagholy et al., 2015).

Over the past decades, neural interfacing systems using various types of transparent and flexible materials have increased in number (Stieglitz et al., 1997; Rodger et al., 2008; Ledochowitsch et al., 2011). To meet the needs of a wide-range of applications, there have been significant advances in graphene/carbon-based neural probes (David-Pur et al., 2013; Du et al., 2015) as well as those based on conjugated polymer systems (Mercanzini et al., 2008; Khodagholy et al., 2016). The advantages of PEDOT:PSS in neural interfacing studies have been previously demonstrated as well, showing improvement in recording quality while reducing tissue reactions (Khodagholy et al., 2011; Someya et al., 2016; Williamson et al., 2015). This mixed conduction material system has received a great deal of attention due to its excellent electronic and mechanical properties, and the resulting practicality and commercial availability (Elschner et al., 2011)_._Additionally, as a result of the success of PEDOT:PSS, a great deal of research has gone into understanding this polymer system and optimizing its mechanical and electrical properties (Crispin et al., 2006; Martin et al., 2010; Proctor et al., 2016; Rivnay et al., 2016). The progress made in this area to date is significant for the field of bioelectronics (Rivnay et al., 2014). Making use of the development and optimization of PEDOT:PSS, electrodes of very small size can be engineered, while retaining low electrochemical impedance and thus maintaining excellent neural recording ability. These properties were taken advantage of here to enable simultaneous optical and electrical characterization of neural networks.

In this study, we were able to show seizure events with electrophysiological recordings and the corresponding optical signals form many neurons (Fig. 3). Comparison was made between optical data that were recorded from the same location as the PEDOT:PSS electrode used to obtain the electrical recording, i.e., Figure 3*A*,*B*. The simultaneous electrophysiological and optical recording strategy could be employed in future applications to extrapolate seizure foci in pathophysiological networks and, using readily available transgenic animals, to identify the exact neuronal cell types behind the pathologic activity using imaging *in vivo*. Moreover, because of the high scalability of this approach, it will be useful to investigate healthy physiologic systems. The size and shape of the of the overall grid are extremely adjustable and may be easily altered depending on the targeted application. In addition, the density of electrode sites may be adapted to optimize recording from specific brain regions. For example, the design and implementation of a grid that could cover the entire visual cortex, or simply particular sub-regions, is straightforward. Whereas previous studies have used relatively large electrode surfaces (on the scale of 50 to several hundred micrometers; Kuzum et al., 2014; Lu et al., 2016), micro-fabricated PEDOT:PSS electrodes easily allow for reduced electrode size to the 10-µm scale while keeping the impedance in the biologically relevant/desired range. Furthermore, although the metal contact lines will become problematic at very high site densities as a result of optical path obstruction and the photoelectric effect, the possibility to stack conduction lines vertically using these materials (Donahue et al., 2017) may be employed to increase site density while retaining high transparency. In addition to the low impedance of PEDOT:PSS electrode sites, which maintains the recording capability for biological signals, these sites can also be used for electrical stimulation (Wilks et al., 2009; Williamson et al., 2015). It could therefore be conceivable to take the advantage of this option of the micro-grid and combine local stimulation with simultaneous 2P imaging to assess signal propagation in neuronal networks. Lastly, as PEDOT:PSS electrodes have demonstrated good biocompatibility (Berggren and Richter-Dahlfors, 2007) as well as the ability to retain recording capability from weeks up to multiple months *in vivo* (Khodagholy et al., 2015; Kozai et al., 2016), this type of probe could also be employed for chronic implantation.

In summary, the present work demonstrates a novel method to perform classical electrophysiology recordings using a state-of-the-art microelectrode array along with 2P imaging of network activity *in vivo*. Initial results demonstrate good correlation of induced pathologic activity using this dual characterization method, providing the best of the two methods: high spatial resolution from 2P imaging combined with high temporal resolution of the multielectrode array. This approach may be developed further to include depth electrodes to simultaneously capture electrophysiological and 2P measurements of single-cell activity across networks.

## Synthesis

Reviewing Editor: Muriel Thoby-Brisson, CNRS UMR 5287 Université Bordeaux Decisions are customarily a result of the Reviewing Editor and the peer reviewers coming together and discussing their recommendations until a consensus is reached. When revisions are invited, a fact-based synthesis statement explaining their decision and outlining what is needed to prepare a revision will be listed below. The following reviewer(s) agreed to reveal their identity: Duygun Kuzum, Kenneth Shepard.

In this paper, the authors develop a mostly transparent microelectrode array, which has potential to be used for concurrent imaging of the underlying neural networks. The reported electrode array demonstrates high transparency owing to Parylene-C substrate and encapsulation. The sizes of the electrodes and wires are restricted to sub-20-μm scale in order to avoid blocking the field of view under two-photon microscope. Miniaturization of the electrodes through surface coating (PEDOT:PSS) and using highly transparent thin film substrates allows these windows to be large and transparent, which is promising to serve as a novel multimodal surface recording array. In vivo experiments are conducted for simultaneous electrical recording and two-photon calcium imaging of chemically induced epileptiform activities.

Although, this study does not focus on any specific neuroscientific research, smart design and precise fabrication of the array with strong practical implications are believed to fit well for the scope of the journal. However, there are major issues need to be addressed before consideration for publication:

Major concerns:

1. The introduction does not provide an overview of transparent electrodes previously demonstrated in various publications including two photon microscopy, optogenetcis or other optical modalities. Instead, it discusses not so relevant glass pipettes. In order to provide a complete view of the topic, the authors should first discuss that literature on previous reports of transparent electrodes made of various materials and then explain how their approach is different in the intro section of the paper.

In addition, the introduction emphasizes the importance of the high spatial resolution PEDOT:PSS coated micro electrodes has to offer. However, imaging through the array requires a certain level of decrease in the electrode density. Did the authors investigate this further with different inter-electrode spacings? What is the maximum array density that would still allow imaging?

The imaging relies on the transparent windows not electrodes. Yet, the introduction and the title should be adjusted to reflect that partial transparency.

2. In Fig. 3C, why is ephys so noisy? I am assuming scale bar is 200uV (typo : 200V) so peak to peak noise is more than 100uV? Isn't PEDOT very low impedance so should have low noise as well? I don't think the recordings in Fig. 3.C look like epileptiform activity at all. There are no clear epileptic spikes or seizures. Could it be an artifact due to laser scanning across the electode or wires? I think this experiment needs to be repeated with proper controls, such as recording ephys without imaging and recording ephys in saline or on a phantom to make sure that these are biological signals but not pure artifacts.

3. The authors mention that the induced photoelectric effect caused by scanning over the metallic electrodes. (Kozai et al., 2015). But there is no data in the paper showing whether PEDOT has or does not have photoelectric artifacts. Even though, the imaging does not occur through the electrodes, considering the non-transparent gold interconnects are in the path of the beam, why does it not cause any photo-electric effect as mentioned in the reference? Is the imaging limited to the inter-electrode windows or is it possible to scan the entire surface? This requires further explanation in the script.

4. The validation of the concept is done through chemically induced seizures. Is this because the local calcium signals are needed to be boosted, as the array covering the brain attenuates them? Were the authors able to record and relate any spontaneous activity?

5. In Line 129, in addition to the pictures of the device, the authors should specify the size of the electrode sites and the spacing of the interconnect wires, because it is not easy for the readers to get the accurate readings from the pictures.

6. The reported 16 electrode array is mostly transparent since the electrodes and wires are small in size and the parylene substrate itself is transparent. However, this claim is only valid for low-density arrays. The authors should clearly mention in the discussion that since the electrodes and wires are not transparent, in high density arrays wires will occupy significant area so they will not have this transparency advantage. Therefore the discussions on scalability need to be revised as well.

Some Other Minor Comments

7. In Line 76, the authors mention that 3-(Trimethoxysilyl)propylmethacrylate is present in the chamber as an adhesion promoter. Please specify the use of this adhesion promoter, whether it was mixed with the dimer of Parylene-C in the vaporizer or coated on the sample substrate. References on this promoter are also highly recommended.

8. In Line 83-88, the authors only mention the thickness of the spin-coated layer of PEDOT:PSS without showing the actual thickness of PEDOT:PSS on the electrode sites of the final device, which is probably different from the former thicknesses. The electrochemical impedance is related to the thickness of PEDOT:PSS, because PEDOT:PSS is a volumetric electrode material. The authors should clealy provide the thickness of PEDOT:PSS. Similarly, in the diagram of Figure 1A, the thickness of the spin-coated PEDOT:PSS layer on the Parylene-C layer seems to be the same as it on the electrode sites, which is misleading because these two thicknesses might be different.

9. The picture of Figure 1E is not clear enough to demonstrate the transparency of the electrode and should have higher resolution. The authors could also use a colorful background for better illustration.

10. In Line 100-101, the format of the unit is not correct, “-1”s should be typed as superscript.

11. In Line 81, “O2/CHF3” should have “2” and “3” as subscript.

12. In Line 87, “140C” should have the symbol “{degree sign}”.
